# Supplementary figures and images for: Strain-Level Diversity Impacts Cheese Rind Microbiome Assembly and Function
Source: mSystems. 2020 Jun 16;5(3):e00149-20. doi: 10.1128/mSystems.00149-20 (PMC7300356; doi:10.1128/mSystems.00149-20)

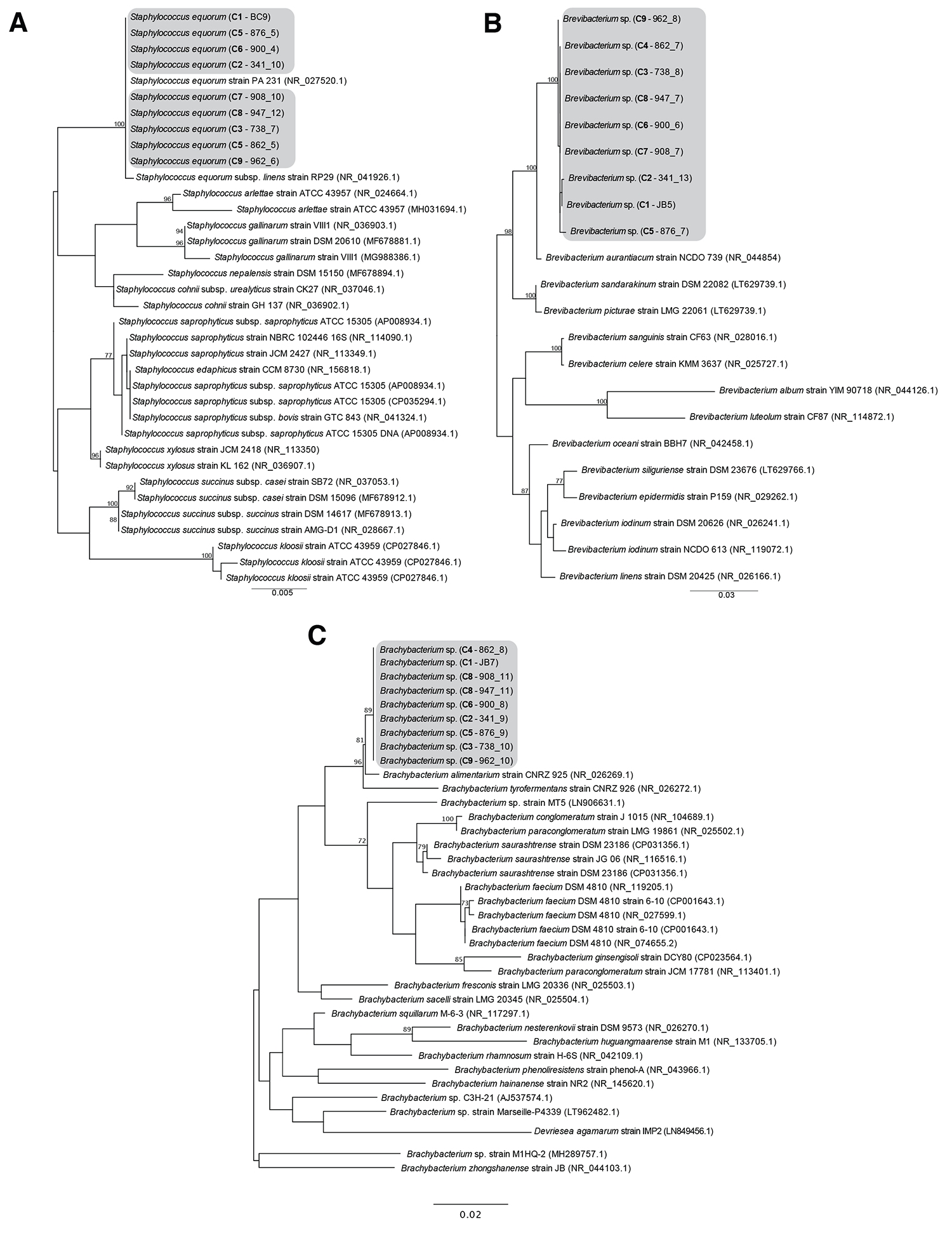

Supplement: FIG S1 [file mSystems.00149-20-sf001.tif]

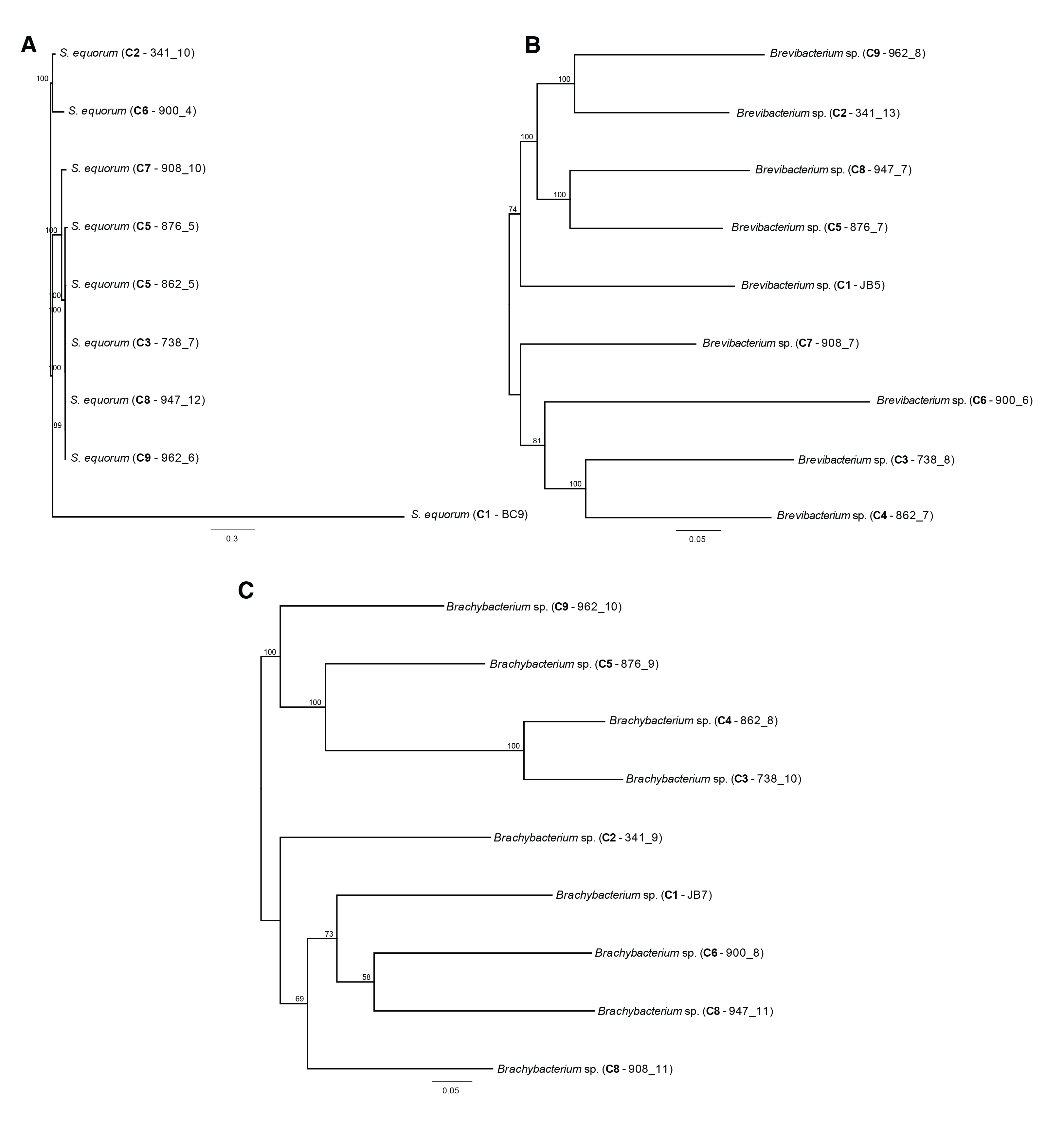

Supplement: FIG S2 [file mSystems.00149-20-sf002.tif]

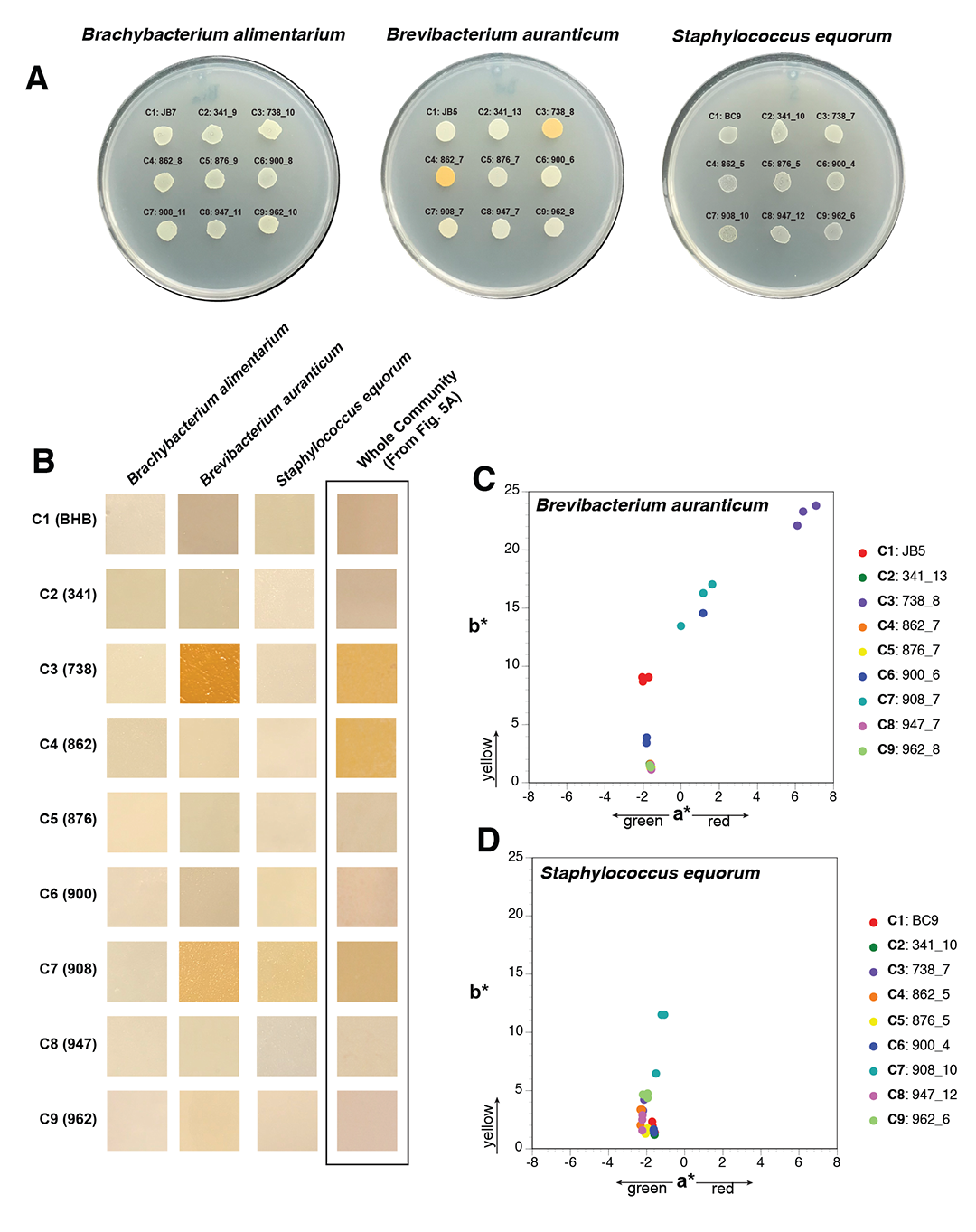

Supplement: FIG S3 [file mSystems.00149-20-sf003.tif]
